# Timing impact of single shot femoral nerve block on rebound pain in patients undergoing total knee arthroplasty: a prospective randomized controlled trial

**DOI:** 10.3389/fmed.2026.1787979

**Published:** 2026-04-10

**Authors:** Meng Ding, Chenqing Zhou, Yujia Tang, Runsheng Huang, Sheng Xu, Jiawei Xiao, Chunmei Liu, Ya Xiao, Xin Wei

**Affiliations:** 1 Department of Anesthesiology, The First Affiliated Hospital of University of Science and Technology of China, Hefei, China; 2 Department of Anesthesiology, Lu'an People's Hospital of Anhui Province, Lu'an, China; 3 Department of Anesthesiology, Geriatric Hospital of Nanjing Medical University, Nanjing, China

**Keywords:** arthroplasty, replacement, knee, femoral nerve block, opioid analgesics, postoperative pain, rebound pain

## Abstract

**Introduction:**

The number of total knee arthroplasties (TKA) is steadily increasing worldwide, exceeding 3 million cases annually. Postoperative pain affects over 60% of patients and is a major barrier to early recovery. Femoral nerve block (FNB) is widely used for analgesia in TKA. This study investigated whether the timing of FNB influences the incidence of rebound pain after TKA.

**Methods:**

In this prospective randomized trial comparing two active interventions, 186 patients undergoing primary TKA were assigned to a pre-FNB group (FNB before surgery using 20 ml of 0.375% ropivacaine) or a post-FNB group (FNB after surgery with the same protocol). The primary outcome was rebound pain within 24 h postoperatively, assessed using the numerical rating scale (NRS). Secondary outcomes included intraoperative anesthetic consumption, nocturnal pain intensity (8–12 h postoperatively), chronic postoperative pain at 3 months, extubation time, post-anesthesia care unit (PACU) stay, Steward score at PACU discharge, number of patient-controlled analgesia (PCA) presses, length of hospital stay, and patient satisfaction before discharge.

**Results:**

Rebound pain occurred in 16.1% (15/93) of patients in the pre-FNB group and 31.2% (29/93) in the post-FNB group (*P* = 0.016; relative risk = 0.52, 95% confidence interval 0.30–0.90). Mean propofol and remifentanil consumption were significantly lower in the pre-FNB group (237.64 ± 99.40 mg vs. 368.98 ± 100.29 mg, and 0.65 ± 0.21 mg vs. 0.97 ± 0.28 mg, respectively; both *P* < 0.001). Nocturnal pain intensity was also lower in the pre-FNB group (*P* = 0.021).

**Conclusion:**

Preoperative FNB significantly reduced rebound pain incidence, lowered intraoperative opioid use, and improved nocturnal pain control compared with postoperative FNB, which may contribute to enhanced recovery.

## Background

Total knee arthroplasty (TKA) is the standard surgical intervention for patients with severe osteoarthritis (1), but over 60% experience intense postoperative pain (2).

Early rehabilitation therapy and functional recovery are essential yet often hindered by pain. Therefore, optimizing postoperative pain management post-TKA is a major clinical priority. Peripheral nerve blocks (PNBs), particularly regional anesthesia, are widely used due to their efficacy in providing postoperative analgesia. However, several studies have shown that once a PNB wears off, pain can rebound suddenly and intensely, a phenomenon known as “rebound pain” (RP) (3, 4). In 2020, Barry Garrett S et al. (5) defined RP as the transition from well-controlled pain (NRS ≤ 3) to severe pain (NRS ≥7) within 24 h of block performance. Unlike general postoperative pain, RP is often more intense, develops rapidly (within 3 to 6 h after the block wears off), and frequently disrupts sleep due to its nocturnal nature. Patients typically describe it as a burning, severe pain, which can result in unplanned analgesic use and a diminished recovery experience (6). RP incidence has been reported to range between 25 and 44%. Although adductor canal block (ACB) is currently the gold standard for regional analgesia in TKA (7). Additionally, the push for faster recovery and shorter hospital stays limits the use of continuous nerve blocks.

Rebound pain following lower limb nerve blocks, including FNB, has been reported (8, 9). While continuous FNB provides effective pain relief, it may compromise quadriceps strength and increase infection risk. In contrast, Single-shot FNB is simpler to perform, cost effective, avoids catheter misplacement, and carries a lower risk of infection. FNB can be administered either before or after surgery. Preoperative FNB may provide preemptive analgesia by blocking nociceptive input before incision, thereby reducing peripheral and central sensitization, but may lead to a shorter duration of postoperative analgesia. Conversely, postoperative FNB may provide prolonged pain relief and potentially reduce RP. A previous study had demonstrated that PNB performed perioperatively offers superior pain control and recovery outcomes compared to general anesthesia alone (10). However, no studies have yet compared how the timing of single-shot PNB affects the incidence of rebound pain. This study aimed to determine whether preoperative or postoperative FNB is more effective in preventing RP in patients undergoing TKA.

## Methods

This study was conducted in accordance with the Declaration of Helsinki. The study protocol was fully reviewed and approved by the Institutional Ethics Committee of The First Affiliated Hospital of University of Science and Technology of China prior to patient enrollment (Approval No. 2022-ky268; Approval Date: October 27, 2022). Written informed consent was obtained from all participants. The trial was registered retrospectively at the Chinese Clinical Trial Registry (ChiCTR2500102391) due to an administrative oversight; however, the protocol followed remained identical to the one approved by the ethics committee.

### Trial design, setting, and population

This single-center randomized trial comparing two active interventions was conducted from late October to December 2022 at the First Affiliated Hospital of University of Science and Technology of China, a tertiary teaching hospital located in Hefei, China. Written informed consent was obtained from all participants before enrollment. Eligible patients were aged 50–79 years, had American Society of Anesthesiology (ASA) status I-III, and were scheduled for primary, unilateral, cemented TKA. To align the 8 to 12-h postoperative assessment with nighttime sleep and the expected window of block resolution, we specifically enrolled patients scheduled for the first two surgeries of the day (typically between 8:00 and 10:00 a.m.). Patients were required to consent to receive FNB for postoperative analgesia and have no history of drug abuse. Exclusion criteria included severe hepatic or renal dysfunction, coagulation disorders, allergy to study medications, puncture site infection, or chronic analgesic therapy history. Patients were randomly assigned to one of two groups using opaque, sealed envelopes: Pre-FNB group (FNB was conducted before anesthesia and surgery), and Post-FNB group (FNB was conducted after surgery). While it was impossible to blind the anesthesiologist and the patients (as the Pre-FNB group received the block awake before anesthesia induction), strict outcome-assessor blinding was maintained. For the Post-FNB group, the block was administered in the PACU while patients were still recovering from general anesthesia and under adequate sedation. Furthermore, the ward nurses who assessed the postoperative scores were completely blinded to the group allocations. The 0.375% ropivacaine used in this study for postoperative analgesia in patients undergoing total knee arthroplasty (TKA) is a standard and approved medication in our hospital. This medicine is commonly used in clinical practice at the First Affiliated Hospital of the University of Science and Technology of China.

General anesthesia was induced using propofol (1–1.5 mg/kg), sufentanil (0.5 ug/kg), and rocuronium (0.6 mg/kg), followed by endotracheal intubation and mechanical ventilation. Anesthesia was maintained with a propofol infusion (4–10 mg/kg/h, sevoflurane (1 MAC), and remifentanil (0.25–0.4 mg/kg/min). Mean arterial pressure (MAP) was maintained within 20% of baseline, depth of anesthesia was monitored using bispectral index (BIS) kept within 40–60. Postoperative patient-controlled analgesia (PCA) included sufentanil (2 μ g/kg), flurbiprofen axetil (100 mg) and Ondansetron (8 mg), diluted in 100 ml of solution. The PCA device was set to continuous infusion rate of 2 ml/h, with a bolus dose of 2 ml, a lockout interval of 20 min, and a maximum of 10 ml per h.

### Surgical and analgesic interventions

All surgeries were performed by the same orthopedic team using a medical parapatellar approach without patellar resurfacing. Local infiltration analgesia (LIA) was administered intraoperatively to all patients using a mixture of 0.25% ropivacaine, 0.3 ml of 0. 1% adrenaline, and 50 ml of saline. This was injected into the periarticular tissues, including the medial and lateral posterior articular capsules, deep tissues around the medial and lateral collateral ligaments, subcutaneous tissue, and wound margins. FNBs were performed using a linear ultrasound transducer probe (4-15 Hz, Navis, Wisonic, China). A single 20 ml injection of 0.375% ropivacaine was administered around the femoral nerve at the proximal femoral triangle. In the pre-FNB group, the block was administered before anesthesia induction, while in the post-FNB group, it was administered upon patients' arrival to the PACU. All blocks were performed by the same experienced anesthesiologist. Patients were required to have an NRS <4 prior to PACU discharge.

### Outcome measurements

The primary outcome was the incidence of RP within 24 h after surgery, defined as a transition from well-controlled pain (NRS ≤ 3) to severe pain (NRS ≥7) specifically recorded at the exact point of sensory block resolution within the 24-h observation window. The Tourniquet duration, the total consumption of opioids and propofol, the time of extubation, the duration of PACU stay, the Steward score, and NRS score after PACU discharge were also recorded. The NRS score was evaluated at 4, 8, 12, 16, 20, and 24 h after surgery, and the highest score occurred during rebound pain. During the first 4 postoperative h, patients were closely monitored in the PACU and early ward phase. Because the FNB was highly active, pain was uniformly well-controlled (NRS ≤ 3) across both groups, and no severe pain spikes (RP events) were observed during this initial period. Chronic postoperative pain was assessed at 3 months after surgery using the numerical rating scale (NRS). The number of patient-controlled analgesia (PCA) manual presses and the length of hospital stay (LOS) were also recorded. Patient satisfaction was evaluated before hospital discharge using a 5-point Likert scale (0 = completely dissatisfied, 5 = completely satisfied).

### Statistical analysis and sample size calculation

Sample size was calculated using PASS 15.0 software (NCSS, LLC. Kaysville, Utah, USA) based on detecting a clinically meaningful 40% relative reduction in RP incidence between groups, which was estimated from previous literature indicating high RP rates following single-shot nerve blocks (5). The calculation used 80% power and a two-tailed alpha of 0.05. A minimum of 186 patients (93 per group) was required. To account for possible dropouts, 200 patients (100 per group) were enrolled. Statistical analyses were performed using IBM SPSS version 25.0 (IBM Corporation, Armonk, NY). Normality of data distribution was assessed using the Shapiro-Wilk test. Variables with normal distribution (e.g., age, BMI, NRS scores) were reported as mean ± standard deviation (SD) and compared using independent *t*-tests. Levene's test was used to assess homogeneity of variance. Non-normality distributed data were expressed as medians with interquartile interval (IQR, 25–75th percentile). Categorical variables were analyzed using Pearson's chi-square test and presented as counts and percentages. For binary outcomes, relative risks (RR) and 95% confidence intervals (CI) were calculated using log-transformed standard errors (Katz method). A *p*-value <0.05 was considered statistically significant.

## Results

**Figure 1:**
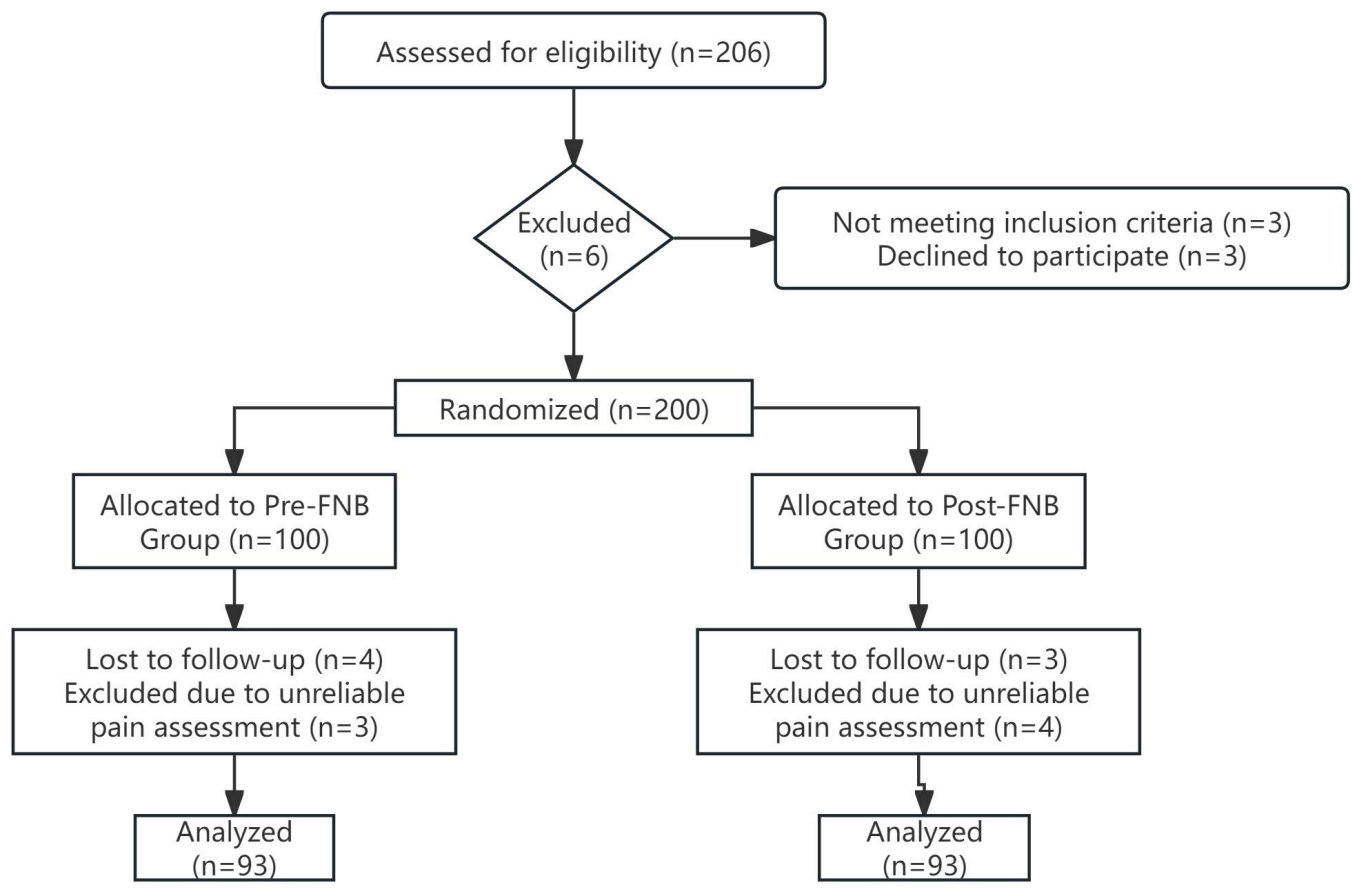
Trial Diagram. CONSORT flow diagram showing the enrollment, randomization, follow-up, and analysis of patients included in the study.

Figure 1 illustrates the Consolidated Standards of Reporting Trials (CONSORT) flow diagram. Between late October and December 2022, of 206 patients initially assessed for eligibility, 6 were excluded prior to randomization. The remaining 200 patients were randomized into the Pre-FNB group (*n* = 100) and Post-FNB group (*n* = 100). Subsequently, 7 patients from each group were lost to follow-up or excluded from the final analysis, leaving 93 participants in each group for the primary analysis.

Table 1 compares the baseline characteristics of the two groups of patients. There were no significant differences between the groups in terms of age, BMI, and chronic pain history.

**Table 1:** Clinical baseline characteristics and perioperative data in the two groups (*n* = 93 each).

Characteristics	Pro-FNB (*n* = 93)	Post-FNB (*n* = 93)	*P*-value
Sex (m/f)	20/73	23/70	–
Surgically affected limb (L/R)	36/57	40/53	–
History of chronic pain (y)	6 ± 5	7 ± 5	0.160
Age (y)	66 ± 7	66 ± 7	0.832
Height (cm)	160 ± 7	159 ± 7	0.407
Weight (kg)	67 ± 11	66 ± 12	0.810
BMI (kg/m2)	26 ± 4	27 ± 4	0.420

Data are presented as mean (SD), median (interquartile range). BMI, body mass index.

**Primary outcome:** As shown in Table 2, the incidence of RP was significantly lower in the Pre-FNB (15/93, 16%) compared to the Post-FNB (29/93, 31%) (*P* < 0.05), the relative risk (RR) of rebound pain for the Pre-FNB group compared with the Post-FNB group was 0.52 (95% CI, 0.30–0.90), indicating a 48% relative reduction in risk.

**Table 2:** Rebound pain and postoperative data in the two groups (*n* = 93 each).

Variables	Pre-FNB (*n* = 93)	Post-FNB (*n* = 93)	*P*-value
Tourniquet duration (min)	67 ± 15	70 ± 14	0.164
Total amount of sufentanil (μg)	28 ± 3	34 ± 3	<0.001
Total remifentanil (mg)	0.65 ± 0.21	0.97 ± 0.28	<0.001
Total propofol (mg)	237.64 ± 99.4	368.98 ± 100.29	<0.001
Rebound pain (*n*)	15/93	29/93	0.016
Highest NRS score in rebound pain	7.8 ± 0.77	8 ± 0.68	0.470
Time to extubation (min)	6 ± 3	11.6 ± 2.7	<0.001
Length of stay in PACU(min)	31 ± 12	41 ± 18	<0.001
Steward score when leaving PACU	5.4 ± 0.8	5.2 ± 0.9	0.357
NRS score when leaving PACU	3.6 ± 0.5	4.0 ± 0.5	0.258
Length of hospital stay (LOS) (days)	5.20 ± 2.25	4.96 ± 2.16	0.776
Patient satisfaction score	3.7 ± 0.4	3.5 ± 0.4	0.263
Incidence of chronic postsurgical pain	5/93	6/93	0.756

PACU, Post-anesthetic care unit; NRS, numeric rating scale.

**Second outcome:** The total dose of administered propofol was significantly lower in the Pre-FNB group (237.64 ± 99.40 mg) than in the Post-FNB group (368.98 ± 100.29 mg) (*P* < 0.05). Similarly, remifentanil consumption was lower in the Pre-FNB group (0.65 ± 0.21 mg) compared to the Post-FNB group (0.97 ± 0.28 mg) (*P* < 0.05) (Table 2). Additionally, differences in sufentanil use and PACU duration were statistically significant (*P* < 0.05).

There were no significant differences between the two groups in terms of Steward score or NRS score upon PACU discharge, the highest NRS score during RP episodes, Length of hospital stay (LOS), or patient satisfaction.

**Figure 2:**
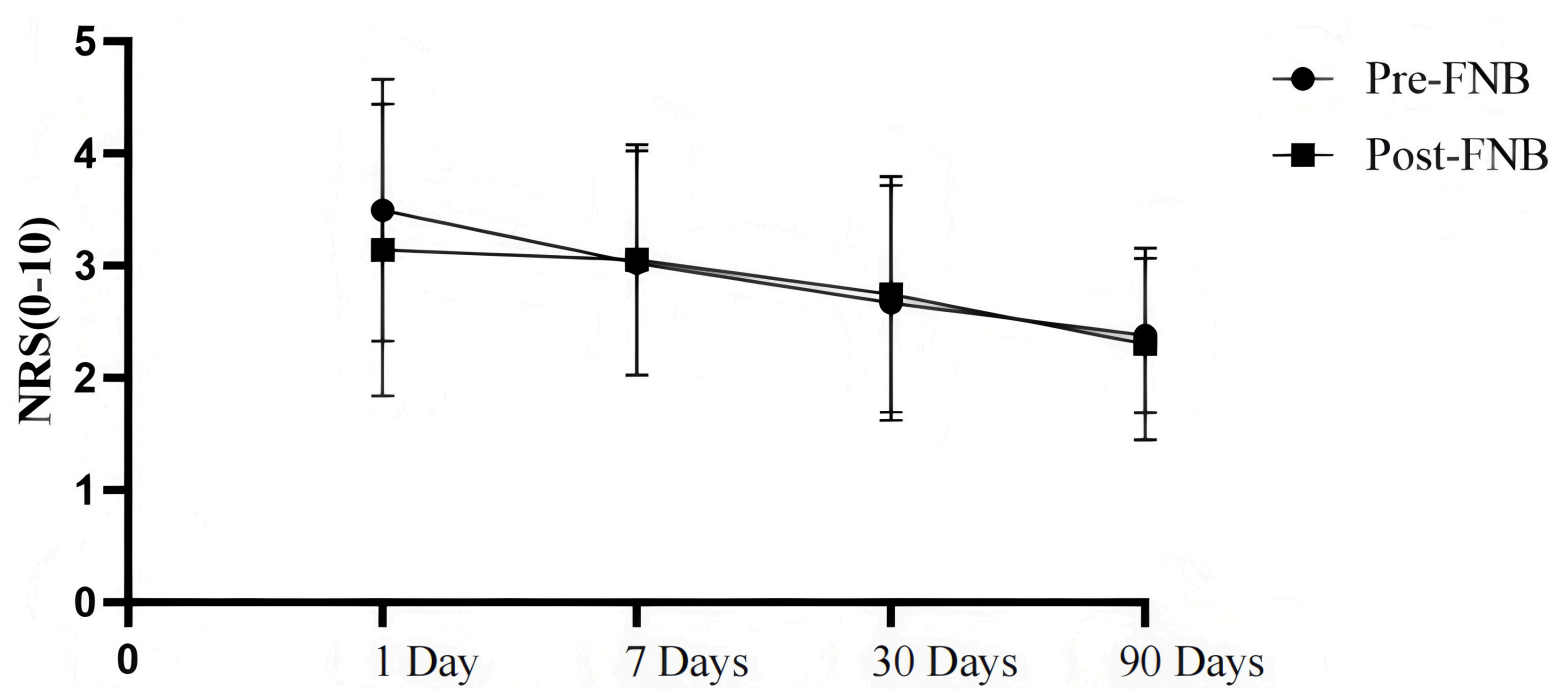
Box plot of pain score changes in patients 24 h after surgery. Box plot of pain score changes in patients 24 h after surgery. Pain scores were evaluated using the numerical rating scale (NRS, 0–10). Data are presented as median (IQR).

Pain intensity was similar between the two groups during the first 4 h postoperatively. However, at 8 and 12 h after surgery, Patients in Pre-FNB group had significantly lower pain scores compared to those in the Post-FNB group (*P* < 0.05) (Figure 2). At these time points, the median NRS pain score was 4.0 (IQR: 3.0-5.0) in the Pre-FNB group vs. 5.0 (IQR: 4.0-6.0) in the Post-FNB group. Notably, both time points fell during the night, as the study only included the first two surgeries of each day. This suggests that preoperative FNB may be more effective in controlling nighttime pain than postoperative FNB.

Table 3 compares patients who experienced RP with those who did not. No significant difference between the two groups was found in gender, age, BMI, total amount of sufentanil, total amount of remifentanil, total amount of propofol, or chronic pain history. However, significant differences were observed in the timing of nerve block administration and the number of manual PCA manual presses (*P* < 0.05).

**Table 3:** Analysis of correlation data between patients with and without rebound pain.

Variables	RP		Non-RP		t/x^2^	*P*-value
	*N*	Percentage	*N*	Percentage		
Number	4	24%	142	76%		
	4					
Gender						
Male	1	25%	32	22.5 %	0.115	0.735
	1					
Female	3	75%	110	77.5%		
	3					
Age (y)	64.84 ± 6.72		66.28 ± 7.38		1.154	0.250
BMI	26.16 ± 4.62		26.40 ± 3.79		0.351	0.726
Sufentanil (ug)	30.98 ± 4.59		31.15 ± 4.77		0.209	0.835
Remifentanil (mg)	0.84 ± 0.39		0.79 ± 0.30		−0.971	0.333
Total Propofol (mg)	333.95 ± 130.25		293.82 ± 114.64		−1.963	0.051
Tourniquet time	1.202 ± 0.25		1.119 ± 0.25		−1.924	0.056
History of chronic pain (y)	6.761 ± 5.37		6.486 ± 5.35		−0.298	0.766
Number of PCA boluses	4.09 ± 1.44		2.06 ± 1.37		−8.447	<0.001
FNB timing						
Pre		15	78		5.835	0.016
Post		29	64			

PCA, Patient-controlled analgesia.

**Figure 3:**
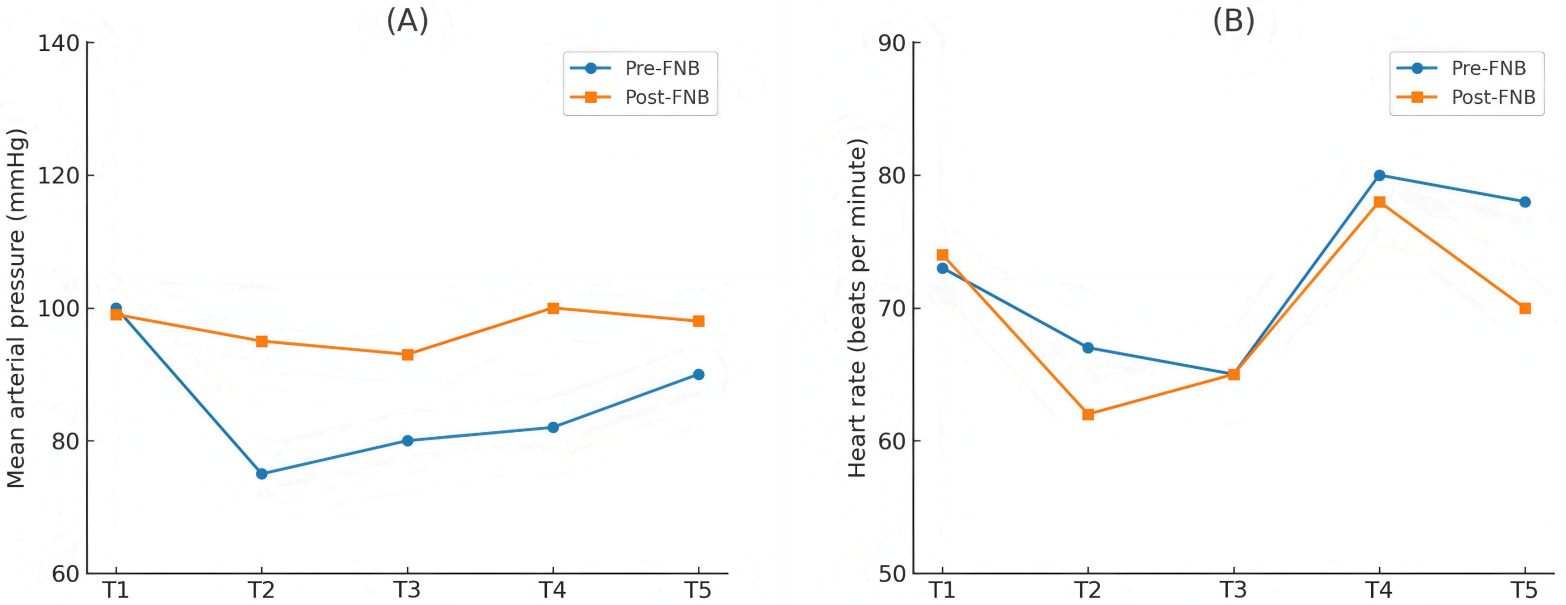
Changes in hemodynamics. **(A)** MAP (mmHg), **(B)** HR (beats per minute); MAP, mean arterial pressure; HR, heart rate. T1, entering the operating room; T2, binding the tourniquet 5 min later; T3, binding the tourniquet 1 h later; T4, releasing the tourniquet 1 min later; T5, entering the PACU 10 min later.

The perioperative changes in mean arterial pressure (MAP) and heart rate (HR) at different time points are shown in Figure 3. The Pre-FNB group exhibited more stable MAP and HR compared to the Post-FNB group.

**Figure 4:**
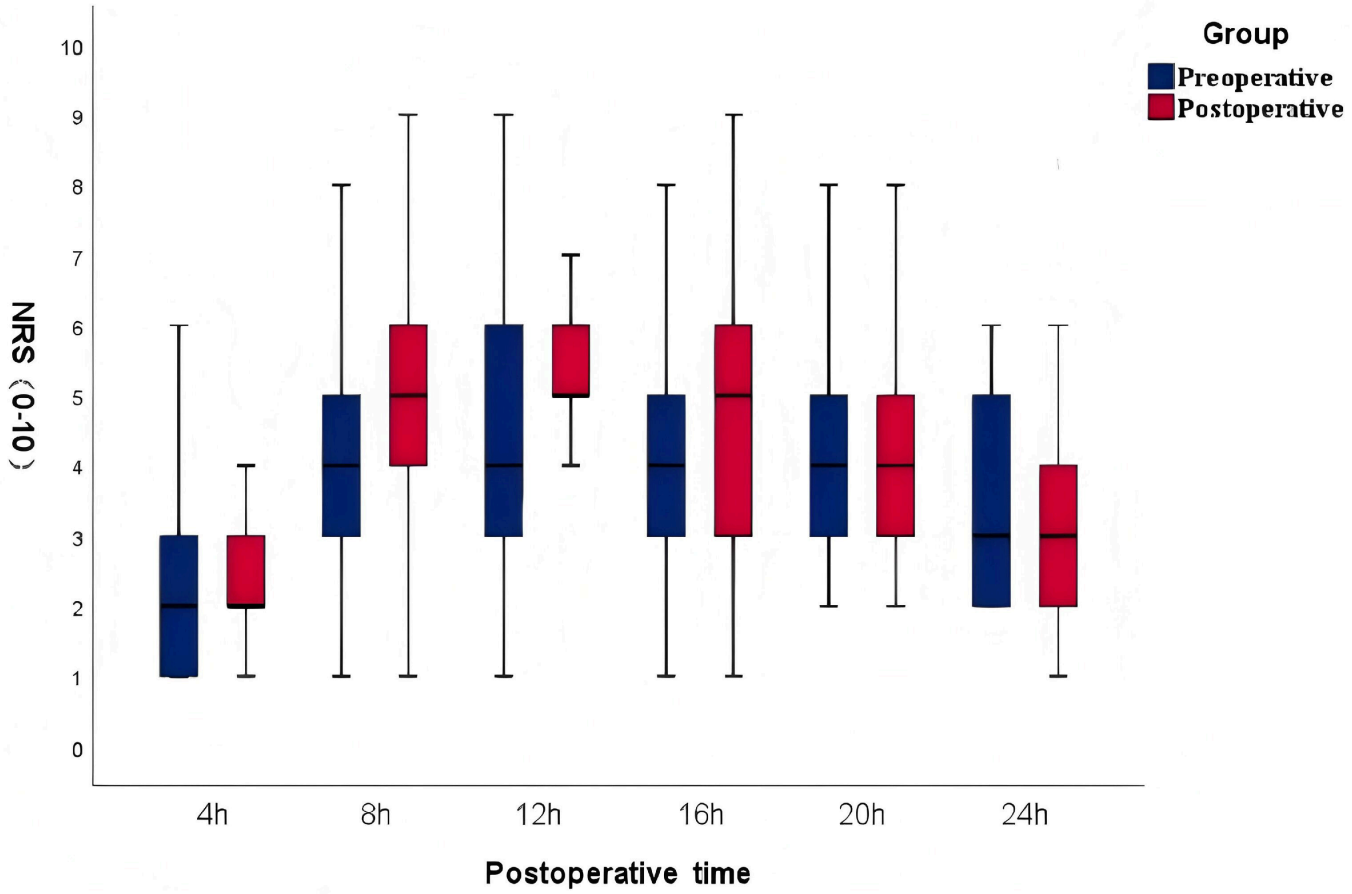
Comparison of pain scores 3 months after operation. PAIN scores were evaluated using the numerical rating scale (NRS, 0–10). Data are presented as median (IQR).

Pain scores at 3 months post-surgery are shown in Figure 4. There were no significant differences between groups at most time points, except at 20 h post-operatively, when the Pre-FNB group reported slightly higher scores than the Post-FNB group (*P* < 0.05). There were no statistical differences between the two groups at other time points.

## Discussion

In this randomized clinical trial, we analyzed 186 patients undergoing total TKA to compare the effects of FNB administered at different time points, preoperatively vs. postoperatively. The primary focus was on the incidence of RP and the pain response within 24 h postoperatively. The results demonstrated that the incidence of RP was significantly lower in the Pre-FNB group compared to the Post-FNB group (16% vs. 31%).

To ensure consistency, we only included the first scheduled surgery each day in single operating room, which meant that the 8-h and 12-h postoperative assessments occurred at night. To minimize confounding factors such as block failure or premature resolution, we excluded patients whose NRS >4 before PACU discharge. The femoral nerve provides the main sensory innervation to the knee joint (11). While continuous nerve blocks and adductor canal blocks (ACB) are often favored for their motor-sparing properties, single-shot FNB remains a valuable technique due to its cost-effectiveness, ease of performance, and reduced risk of catheter-related complications, making it a practical alternative (12). Our study specifically utilized single-shot FNB to isolate the effect of timing on rebound pain mechanisms. Furthermore, we deliberately chose to use plain 0.375% ropivacaine without adjuvants (such as dexmedetomidine or dexamethasone). This was to purely isolate the effect of timing on central sensitization and rebound pain, avoiding confounding variables like unpredictable block prolongation or systemic absorption effects associated with adjuvants. The findings suggest that preoperative administration may mitigate central sensitization early on, a benefit that likely extends to other block techniques as well. Interestingly, our findings suggest that even though the Post-FNB group received the block in a timely manner, they experienced higher rates of RP and worse nocturnal pain control compared to the Pre-FNB group. Several mechanisms may explain this difference. First, from a surgical trauma perspective, administering nerve block preoperatively may inhibit the pain transmission pathway early and raise the pain threshold. Second, patients who received Pre-FNB may have had more relaxed periarticular muscles, reducing iatrogenic soft tissue injury during surgery. Additionally, tourniquet application, often associated with ischemia-reperfusion injury, may exacerbate postoperative pain. Tourniquet inflation leads to intracellular acidosis, ATP depletion, and calcium overload, all of which damage tissues (13, 14). Preoperative FNB may help mitigate this response by blunting the tourniquet-induced stress (15).

Managing nocturnal pain following TKA is crucial. As the effect of nerve block diminishes, the release of pain-promoting substances from injured tissues can trigger significant discomfort, impair immune and endocrine functions, and delay wound healing. Our comparison of NRS scores at 8 and 12 h postoperatively showed significantly lower pain levels in the Pre-FNB group, indicating better control of nighttime pain. While no significant differences in pain scores were observed between the groups at 3 months postoperatively, the NRS score at 24 h was slightly higher in the Pre-FNB group. This may be due to earlier block resolution or more frequent PCA usage in the Post-FNB group. Although various risk factors for RP exist.

Although various risk factors for RP have been reported, including young women, orthopedic surgery, and prolonged tourniquet use (5), this study population primarily consisted of elderly women undergoing similar procedures with comparable tourniquet durations, which likely minimized variability. No significant differences in sedative and analgesic consumption were observed between patients with and without RP. This may be due to the greater intraoperative drug usage in the Post-FNB group, balancing out the potential differences. Both groups received general anesthesia under BIS guidance (maintained at 40–60), and inhalation of sevoflurane was standardized. Interestingly, the Pre-FNB group required significantly less propofol and remifentanil during surgery. This is supported by hemodynamic data showing more stable heart rates and blood pressure in the Pre-FNB group, an important consideration for elderly patients with comorbidities such as coronary heart disease. Although remifentanil can contribute to hypertension intraoperatively, this is dose- and timing-dependent (16, 17). Blood pressure fluctuations exceeding 35% of baseline have been associated with increased risk of post-operative stroke (18).

The pathophysiology of RP remains poorly understood. Some evidence suggests that local anesthetics may induce nerve injury with pro-inflammatory properties, leading to neuropathic pain after peripheral nerve blocks (19). Postoperative hyperalgesia has also been proposed as a mechanism (20, 21); remifentanil use is known to contribute to this phenomenon (22). When opioid drug infusions stop, previously painless injury sites can become painful, a phenomenon known as withdrawal-related injury site pain (23). Therefore, minimizing opioid use may not only accelerate recovery but also reduce RP. While PCA was employed in this study and overall patient satisfaction was high, the analgesic effect was not optimal. The most effective strategy for reducing chronic pain protection and improving functional recovery remains prolonged epidural analgesia starting before surgery and extending several days postoperatively (24, 25). Previous studies have reported RP incidence as high as 50% following single-shot nerve blocks (7). However, no studies to date have explored the effect of neuraxial anesthesia on RP.

This study has some limitations. First, we lacked objective measures to monitor intraoperative pain intensity. Second, all patients received general anesthesia. This was strictly chosen to standardize the intraoperative depth of anesthesia and perfectly control the exact timing of FNB administration without the overlapping variable sensory blockade of spinal anesthesia. However, we acknowledge that this limits the generalizability of our findings to institutions that primarily utilize neuraxial anesthesia for TKA. Third, strict patient blinding was not feasible in our study design. Patients in the Pre-FNB group received the block while awake before anesthesia induction, whereas the Post-FNB group received it while sedated in the PACU. We acknowledge that this lack of patient blinding introduces a major potential source of bias regarding subjective pain reporting. Fourth, this study utilized single-shot FNB primarily as a mechanistic model to investigate the impact of intervention timing on RP. We acknowledge that current clinical guidelines, including the 2022 Chinese Guidelines for TKA, recommend motor-sparing techniques (e.g., adductor canal block combined with IPACK) over FNB to avoid quadriceps weakness. While we do not advocate for routine FNB use in modern TKA protocols, our findings provide a proof-of-concept that preemptive neural blockade effectively mitigates central sensitization compared to post-incision administration. Future studies should explore whether this preoperative timing advantage applies to modern motor-sparing techniques. Future prospective studies comparing different anesthesia modalities, such as spinal anesthesia vs. PNB, are warranted to further elucidate their effects on RP.

## Conclusion

Compared to postoperative FNB, preoperative FNB significantly reduces the incidence of postoperative RP, decreases intraoperative opioid consumption, and improves nighttime pain control in patients undergoing TKA. These benefits suggest that preoperative FNB may offer a more effective strategy for enhancing recovery and pain management in TKA patients with similar characteristics and in comparable clinical settings.

## Data Availability

The raw data supporting the conclusions of this article will be made available by the authors, without undue reservation.
